# EFFECTS OF MENOPAUSE MODELING ON AUTOPHAGIC AND LYSOSOMAL FUNCTION IN ALZHEIMER’S DISEASE MICE

**DOI:** 10.1093/geroni/igae098.2586

**Published:** 2024-12-31

**Authors:** Danielle Osborne, Adrianah Dorn

**Affiliations:** Legacy Research Institute, Portland, Oregon, United States; Legacy Research Institute, Portland, Oregon, United States

## Abstract

Alzheimer’s disease (AD) is denoted by strong sex differences, whereby females are significantly more likely to develop AD and often experience more rapid progression and cognitive decline. This difference in risk is often attributed to menopause, however the underlying molecular mechanisms are unknown. In humans and animal models, autophagic and lysosomal dysfunction are among the earliest known cellular changes that occur in AD susceptible brain regions, like the hippocampus. Our goal was to first characterize how key autophagic and lysosomal gene transcripts changed due to sex, ovariectomy (OVX) and estrogen replacement in wildtype mice, then investigate in female AD mice how these cellular processes change due to OVX and estrogen replacement. In wildtype mice, across 22 genes investigated, three transcripts, Atg12, Atg5, and Map1LC3b showed a baseline sex difference. Using the 5xFAD model of AD, we observed the expected genotype difference in cognition, with transgenics having a significant impairment in Morris watermaze performance. Probe measures indicate decreased performance due to OVX, but that 5xFAD mice may be further hindered by the addition of estrogen. Molecular measures will include western blot analysis of phosphorylated proteins involved in autophagic/lysosomal signaling (pAkt, pAMPK, pRPS6), and qPCR (Atg5, Atg12, Map1LC3b, Tfe3, Tfeb, among others) in the hippocampus, and immunohistochemistry (GFAP, Iba1, amyloid-beta, reelin) in the hippocampus and entorhinal cortex. These studies will provide valuable information on how cessation of female hormones at menopause affect the vital cellular functions of autophagy and lysosomal degradation in key cognitive and disease susceptible brain regions in AD.

